# Telemedicine and patients with heart failure: evidence and unresolved issues

**DOI:** 10.31744/einstein_journal/2024RW0393

**Published:** 2024-02-19

**Authors:** Tarso Augusto Duenhas Accorsi, Gabriela Guimarães Rodrigues dos Santos, Renato Paladino Nemoto, Flavio Tocci Moreira, Karine De Amicis, Karen Francine Köhler, Eduardo Cordioli, Carlos Henrique Sartorato Pedrotti

**Affiliations:** 1 Hospital Israelita Albert Einstein São Paulo SP Brazil Hospital Israelita Albert Einstein, São Paulo, SP, Brazil.

**Keywords:** Heart failure, Morbidity, Mortality, Telemedicine, Telecare

## Abstract

Heart failure is the leading cause of cardiac-related hospitalizations. Limited access to reevaluations and outpatient appointments restricts the application of modern therapies. Telemedicine has become an essential resource in the healthcare system because of its countless benefits, such as higher and more frequent appointments and faster titration of medications. This narrative review aimed to demonstrate the evidence and unresolved issues related to the use of telemedicine in patients with heart failure. No studies have examined heart failure prevention; however, several studies have addressed the prevention of decompensation with positive results. Telemedicine can be used to evaluate all patients with heart failure, and many telemedicine platforms are available. Several strategies, including both noninvasive (phone calls, weight measurement, and virtual visits) and invasive (implantable pulmonary artery catheters) strategies can be implemented. Given these benefits, telemedicine is highly desirable, particularly for vulnerable groups. Although some questions remain unanswered, the development of new technologies can complement remote visits and improve patient care.

## INTRODUCTION

Heart failure (HF) is a highly prevalent condition in adults and is associated with substantial morbidity and mortality. Its prevalence in the European population is estimated at 0.4%-2%.^(1)^ In the United States, the number of individuals with HF is expected to increase to 8.4 million people by 2030.^(2)^

Despite significant advances in knowledge regarding pathophysiology, diagnostic tests, and treatments, HF remains a complex and progressive syndrome that places a huge burden on patients and health systems.^(2)^ Several treatment options are available, and frequent reassessments are essential. Unfortunately, limited access to outpatient appointments restricts the application of guideline-directed and patient-tailored medical therapy, resulting in HF being the most common cardiac-related cause of hospitalization and re-hospitalization, with a mortality rate similar to that of acute coronary syndrome.^(3–5)^

Therefore, it is imperative to adopt strategies that prevent rapid deterioration in HF (A-D) stages, *i.e*., emphasizing the control of risk factors, correct prescription, and adherence to pharmacological and behavioral treatment.^(6)^

**Figure 1:**
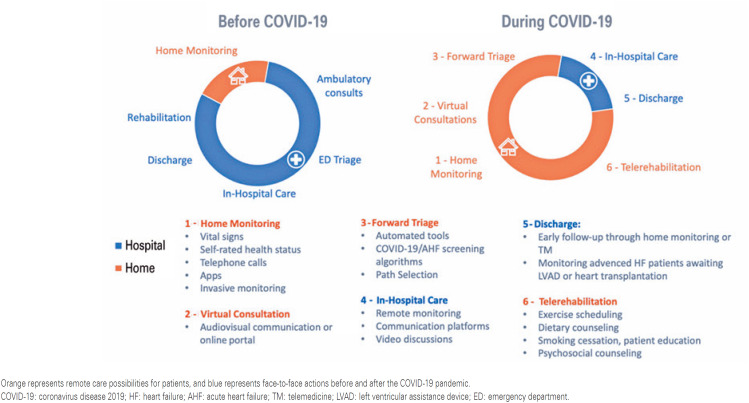
Changes after the COVID-19 pandemic

**Figure 2:**
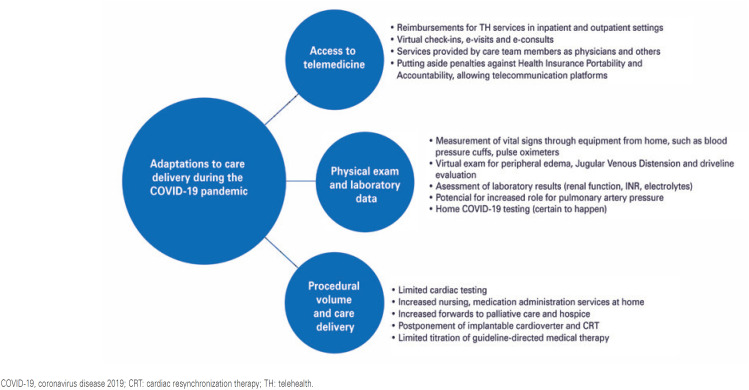
Adaptation to care after pandemics involving telemedicine. Key aspects of telemedicine

Telemedicine (TM) has become an essential resource in the healthcare system because of its cost-effectiveness, as it can safely reach large populations through prompt actions. In 2020, the coronavirus disease 2019 (COVID-19) pandemic exponentially accelerated the development of virtual visits.^(7)^ Telemedicine allows multiple interactions between patients and healthcare providers through teleguidance, telemonitoring, teleconsultation, or direct-to-consumer evaluation.^(5)^ Telemedicine has great potential in medical care to allow more effective actions in prevention, diagnosis, treatment, and follow-up^(8)^ (Figures 1 and 2).

This narrative review aimed to demonstrate the evidence and unresolved issues regarding the use of TM in patients with HF.

### Key aspects of telemedicine

The first article describing TM was published in 1974 when Murphy et al described 1000 patients evaluated from August 1968 to December 1969 in a medical facility at Boston International Airport through remote nursing consultations using an ancient internal television circuit connected to the Massachusetts General Hospital, three miles away.^(9)^ This pioneering study demonstrated the feasibility of physical examinations performed using video, with high diagnostic accuracy in many situations and high patient satisfaction. Since then, there has been an impressive growth in the number of publications on TM in different areas of knowledge, geographical situations, and social conditions. Currently, majority of patients have access to audio and video devices, and can work alone or with the assistance of people close to them.^(10)^ In May 2005, Ministers of Health from 192 member countries of the World Health Organization (WHO) approved the Resolution on e-Health, which recognized, for the first time, the importance of information and communication technologies applied to health-digital health or eHealth-"reinforcing the fundamental human rights by increasing and improving equity, solidarity, quality of life, and quality of care."^(11)^

Furthermore, the COVID-19 pandemic has boosted TM worldwide and consolidated it as a fundamental strategy in healthcare systems.^(7,12)^ Among the advantages and possibilities of TM are remote assistance (teleconsultation, telediagnosis or diagnostic telemonitoring, remote patient monitoring, and treatment), administrative management of patient care (request for diagnostic tests, medical prescriptions, and actions related to service reimbursement), remote qualification of human resources to facilitate continuing education programs, and worldwide collaboration in clinical research (sharing and disseminating best practices and generating knowledge; Table 1).^(8)^ Additionally, TM provides an opportunity for teleconsultation with specialists and professionals, allowing patients to receive a comprehensive therapeutic approach at home.^(13,14)^

**Table 1:** Benefits of telemedicine

Potential benefits of telemedicine in heart failure
Maintain face-to-face interaction with less exposure
Involve caregivers and family
Provide access
Maintain connection with patient
Faster titration of medications
Reduce consultation intervals
Vital signs and hemodynamic status
Prevent decompensation
Rehabilitation
Enhance adherence

Source: Adapted from Gorodeski EZ, Goyal P, Cox ZL, Thibodeau JT, Reay RE, Rasmusson K, et al. Virtual visits for care of patients with heart failure in the era of COVID-19: a statement from the Heart Failure Society of America. J Card Fail. 2020;26(6):448-56.^(13)^

The most prevalent diseases with the highest morbidity and mortality rates have been addressed using TM, particularly in the last decade. The best evidence of the role of TM was observed in cancer care (tele genetics, remote chemotherapy supervision, symptom management, survivorship care, palliative care and enrolment in clinical trials),^(15)^ diabetes management (retinal screening, glycemic monitoring),^(16)^ neurological disorders (early specialist access, monitoring, rehabilitation, education),^(17)^ dermatology (early specialist access, noninferior remote diagnosis),^(18)^ psychiatry (noninferior evaluation, monitoring)^(19)^ and emergency medicine (on-time specialist consultation, procedures guidance, referral optimization).^(20)^ In addition, pediatric surgery^(21)^ obstetrics and gynecology ^(22)^ perioperative assessment,^(23)^ gastroenterology,^(24)^ allergy^(25)^ and rheumatic diseases^(26)^ have shown some evidence of benefits related to remote evaluation. However, the general impression is non-inferiority regarding anamnesis, orientation, and relationship in remote evaluations compared with physical consultations.^(27)^

Several studies have addressed essential issues such as regulation,^(28)^ ethics,^(29,30)^ implementation strategy,^(31)^ technology,^(32)^ payment^(33)^ and insurance^(34)^ in TM.

Telemedicine plays a role in preventing rehabilitation.^(35)^ Regarding HF, physicians can maintain face-to-face interactions, recognize patients’ domestic environments, obtain vital sign measurements, perform limited physical examinations, and titer medications.^(36)^

A rehabilitation program conducted at home with telephone contact with the rehabilitation team is an option for patients discharged from hospital after an acute event.^(14,37,38)^ Moreover, TM is a tool that facilitates the involvement of patients’ caregivers, which is a central aspect of self-care practice necessary in the HF context. Some patients may find it easier to discuss complex topics in their homes along with their family members, who may not be present during physical consultations.^(13,36)^

### Prevention of heart failure

Several studies have aimed at preventing and reducing HF decompensation. However, no study has specifically addressed the use of TM for the prevention of HF. This is expected owing to extended follow-up, logistics, and financial issues. However, some indirect evidence may be considered, as several studies have shown that telemonitoring strategies are associated with better hypertension control than traditional in-office medical control. In the TASMINH4 study, McManus et al. showed that self-monitoring in patients with telemonitoring orientation yielded faster blood pressure control than the self-monitoring strategy alone, and both were better than traditional medical control.^(39)^ Margolis and al. demonstrated greater long-term blood pressure control by using pharmacist telemonitoring.^(40)^ The INTERACT randomized study showed that text messages used during follow-up were associated with a 16% improvement in adherence.^(41)^ This evidence may be related to the further reduction of cardiovascular events, such as HF manifestation, due to the long-term best control.^(42)^

Regarding lifestyle modifications to prevent HF, some evidence exists regarding weight control, exercise, nutrition (including dietary composition and supplements), and meditation to manage stage A.^(43)^

As explained in the key aspects of TM, a higher level of proximity between the health professional and patient is associated with a higher therapeutic target achievement rate. Similar to hypertension, *diabetes mellitus* is associated with the onset of HF and better management via TM may be a more effective preventive strategy.^(44)^

The Framingham Heart Study revealed that incidence of parental HF increases the risk of heart failure in the offspring.^(45)^ Telegenetics consultation may provide an opportunity to clarify possible risk factors among families that include parents with HF.^(46)^

Finally, if properly managed, patients with chronic renal failure may show less progression to HF if properly managed, which can be enhanced by TM.^(47,48)^

### Telemonitoring of patients with heart failure

Heart failure requires close and intensive outpatient monitoring. Telemedicine can be used to evaluate all patients with HF, including those with preserved or reduced ejection fractions at all stages, those with left ventricular assist devices, and heart transplant recipients.^(38,49)^

Several available platforms make consultation possible and practical. However, the optimal platform depends on the patient’s clinical status, technological awareness, institutional factors, capacities, and objectives.

The available strategies can be either noninvasive or invasive. The non-invasive strategy comprises phone calls, weight measurements, virtual visits, and reinforcement of educational materials. This invasive strategy involves devices that transmit several pieces of information to a remote server, (discussed below).^(50,51)^

The first consideration is to identify the most suitable patients for a virtual visit and retrieve those who are better served by formal consultations.^(49)^

Clinical assessment can include the evaluation of clinical status, medication review, screening for adverse events, up titration, and counselling about medication adherence, diet, exercise, and other non-pharmacological approaches. Additionally, a review of medications might be better in TM because pill bottles are available at home, and the patient or caregiver can demonstrate how he/she is taking them^(49,51)^ which can minimize medication non-adherence, especially after hospital discharge and changes in prescription.^(52,53)^ Moreover, patients near stage D and those who require inotropes can benefit from the potential for decompensation.^(38)^

Weight monitoring is another critical feature that is amenable to remote control and decompensation prediction, indicating early intervention.^(54,55)^ This kind of monitoring is effective when performed by nurses, pharmacists, and physicians.^(56,57)^

**Figure 3:**
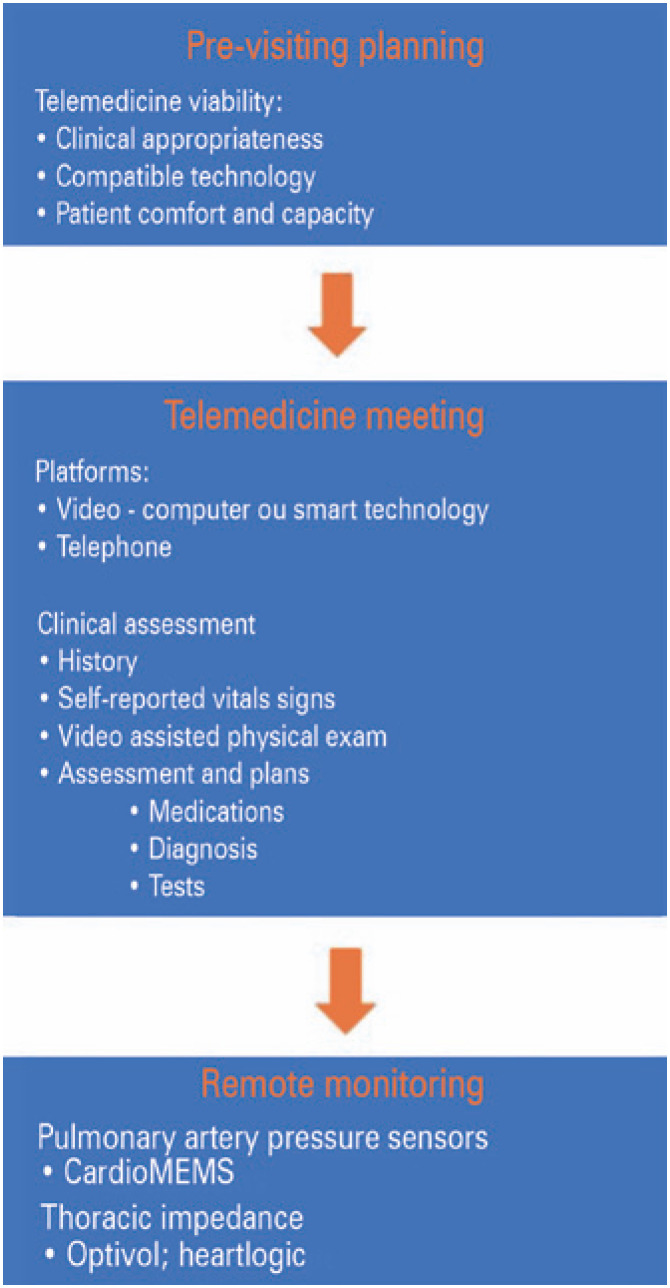
Medical care model for telemedicine consultations. Heart failure treatment and telemedicine randomized controlled trials

The essential components of the physical examination can be performed mainly when patients use high-quality video equipment (available on smartphones and tablets). Alertness and orientation can be assessed, and the volume status can be evaluated by observing signs of peripheral edema (leg swelling and jugular stasis). Orthopnea and bendopnea may also be assessed remotely, both of which are associated with elevated ventricular filling pressure.^(58)^ The evaluation of exercise intolerance may be evaluated by asking the patient to walk from room to room or up a flight of stairs. Peripherally inserted central catheter line sites, other cannulas, and healing surgical incisions such as pacemaker or implantable cardiac defibrillator implantation sites can also be evaluated (Figure 3).^(35,38)^

There is a low level of evidence-based medicine through randomized controlled trials, with most meta-analyses and systematic reviews derived from observational studies.

Almost all evidence of TM in patients with HF is related to the avoidance of hospitalization through orientations and interventions. Majority of controlled trials involve monitoring and consequent treatment adaptation. This interventional management model is superior to conventional care.^(59)^

A Cochrane systematic review of 41 studies on telephone support monitoring of patients with HF observed that this strategy was associated with a 34% reduction in all-cause mortality and 20% HF-related hospitalization.^(60)^ This is consistent with the findings of another meta-analysis carried out by Anker et al.^(61)^ A meta-analysis of vital sign monitoring did not demonstrate a reduction in hospitalization but observed some mortality benefits.^(62)^ A study that followed more than 200 patients with HF and a reduced ejection fraction over 18 months demonstrated a low rate of admissions due to decompensation without an increase in mortality.^(34)^ Additionally, no differences in rehospitalization or mortality were observed in a prospective study.^(63)^

A meta-analysis by Zhu et al. included 29 randomized controlled trials that showed positive results. Telemedicine was associated with a reduction in the total number of all-cause hospitalizations (odds ratio [OR], 0.82; 95% confidence interval [CI], 0.73-0.91; p=0.0004), cardiac hospitalizations (OR, 0.83; 95%CI: 0.72-0.95; p=0.007), and all-cause mortality (OR, 0.75; 95%CI: 0.62-0.90; p=0.003). Additionally, HF-related mortality was similar to that in traditional care (OR, 0.84; 95%CI: 0.61-1.16; p=0.28).^(64)^

In a randomized trial that evaluated the uptitration of carvedilol in 49 patients (remotely and conventionally), the final dose was the same; however, patients in the TM group reached that dose in half the time (33 *verus* 67 days).^(65)^

### Cost-effectiveness

There is strong evidence of cost savings associated with TM adoption compared to that with similar physical evaluations.^(66,67)^

For example, implementing a telemonitoring program can result in a reduction of up to €45.186 per patient or 24% of the total cost, along with an increase in quality of life.^(68,69)^

### Unresolved issues and future challenges and opportunities

To achieve all the above benefits, patients must be willing and the required technology must be available. However, these factors can come with some challenges, particularly for the most vulnerable populations. Nevertheless, the integration of technology within an institution’s electronic portal or app, which is already familiar with the patients, could be an option to overcome barriers.^(35,38)^

Some patients may initially be reluctant or uncomfortable with video interactions, or might have concerns that something is lost without proximity. However, these concerns may become less common as virtual consultations become mainstream.

The privacy of data is paramount and is widely discussed, along with the costs and models of reimbursement.

After the pandemic, TM can be integrated into the care process. Programs may benefit from the establishment of triage principles for stable patients. Heart transplant recipients on stable immunosuppression at a low risk of allograft rejection and hemodynamically optimized patients with LVAD may also be managed remotely, all coordinated by a multidisciplinary team. Physical consultations could only be considered for recently hospitalized patients and those approaching Stage D, who are on continuous inotropes, undergoing evaluation for advanced HF therapies, and are newly post-LVAD or heart transplant implantation. The interaction between hospitals should be more accessible and patients could be evaluated by specialists far away.^(35)^

One of the main issues regarding TM is the limited physical examination. Many existing and emerging diagnostic technologies and wearable devices can address these gaps.^(38)^

Wearables are electronic devices, such as clocks, shirts, contact lenses, and shoes. These devices have sensors that can obtain and send information remotely in real-time through a cloud or server.^(50)^

These technologies rely on the principle that subtle changes in cardiac physiology precede overt signs and symptoms of heart failure and, if used effectively, can prevent hospitalization. Currently, there are two types of monitoring systems: implanted sensors designed to monitor intracardiac filling pressures and cardiac implantable electronic devices (CIEDs) that can measure other physiological parameters.

Several of these methods are currently used. The most basic requirement is the remote monitoring of weight and blood pressure using electronic scales and blood pressure cuffs.^(38,49)^

The cardioMEMS device is an implantable pulmonary artery pressure sensor. It is indicated for patients with a New York Heart Association class III functional capacity and at least one hospitalization within the previous 12 months.^(70)^ It works by monitoring the pulmonary artery pressure changes that are transmitted wirelessly to a website. These data can guide the adjustment of diuretic and vasodilator therapies. Each patient has a preset range of acceptable pulmonary artery pressures, specifically, the target pulmonary artery diastolic pressure. The CardioMEMS system can identify patients at risk for heart failure decompensation and reduce hospitalizations.^(70)^

Certain CIEDs can perform serial thoracic impedance measurements, which are inversely related to the volume overload. As the pulmonary fluid increases, the intrathoracic impedance decreases. The Optivol system (Medtronic) utilizes an algorithm that derives an individualized ‘Fluid Index’ or impedance threshold with these measurements, at which a patient is at risk for decompensation. It is more sensitive than other clinical markers of HF.^(71)^

HeartLogic (Boston Scientific) uses a multisensor algorithm detected using a CIED. This algorithm analyzes five metrics-intrathoracic impedance, nocturnal heart rate, presence of a third heart sound, respiration rate, and patient activity-and creates a quantitative and objective assessment of the clinical state. HeartLogic predicted decompensation events with a sensitivity of 70%. Notably, nearly 90% of patients enrolled in the study had an alert that preceded a true HF event by at least 2 weeks.^(72)^

The available adjuvant sensors are underutilized. This may be partly due to the opportunity for direct physical examination, costs, and time required to set up and maintain a robust remote monitoring platform and process. In addition, other and more compact options still need to be developed to represent an enormous field of interest. Nevertheless, the potential utility of these technologies cannot be underestimated, and when used appropriately, they can be powerful tools in remote management and enhancing the quality of life of patients with HF.^(43,70,73–75)^

There are still no recommendations in the current guidelines for these devices owing to few studies, and the relevant aspect of cost-effectiveness is still unclear.^(2,8,13)^ Moreover, most innovations are unavailable in developing countries.

### Special populations

The use of TM is highly desirable, especially for vulnerable groups. However, disadvantaged populations, who could benefit more from the expansion of these innovations are less likely to be reached.^(13,35)^ Many patients with HF, especially older adults and those living in rural communities, often have difficulty attending visits because of poor exercise tolerance and difficulties with transportation and oxygen, among other barriers.^(38)^

The older population is significant because more than half of the patients currently living with HF in the US are aged >70 years. In addition, recent data have shown that an increasing number of older adults possess smartphones and can be taught to use new technologies.^(38)^

Burdese et al. demonstrated significant benefits in many aspects in a population aged 80 years. There was a reduction in rehospitalization, ER visits, and the cost of support. All the patients had a negligible rate of treatment discontinuation.^(76)^ Antonicelli et al. associated telemonitoring with an improvement in the composite endpoints of mortality and rate of hospitalization, more frequent use of beta-blockers and statins, lower total cholesterol levels, and a better-reported health perception score. This was probably due to better compliance and closer monitoring of patients, which was enhanced by distance monitoring.^(77)^ On the other hand, a randomized control trial did not show the benefits of a telehealth system follow-up compared to ‘traditional’ follow-up in a population of 164 patients with an average age of 78 years.^(78)^

An important consideration is that even after the COVID-19 crisis ends, patients may continue to have concerns about in-person office visits and travel and may prefer to continue social distancing. As a result, many patients with HF may continue to prefer virtual consultations.

## CONCLUSION

Telemedicine has emerged as an essential tool for managing and caring for patients and plays a unique role in the population with heart failure. It aids in close monitoring and prevention of decompensation, facilitates access to consultations, and brings distant people closer. With technological development, more compact and robust devices will be designed to complement remote visits and improve patient care. The impact on the morbimortality of telemonitoring of patients with heart failure with cutting-edge technology needs to be better studied.
